# Revealing the mechanism of extraordinary hardness without compensating the toughness in a low alloyed high carbon steel

**DOI:** 10.1038/s41598-019-55803-6

**Published:** 2020-01-13

**Authors:** Rumana Hossain, Farshid Pahlevani, Veena Sahajwalla

**Affiliations:** Centre for Sustainable Materials Research and Technology, School of Materials Science and Engineering, UNSW Sydney, Sydney, Australia

**Keywords:** Metals and alloys, Mechanical properties

## Abstract

There is a continuous quest for discovery of a steel grade which has better properties and lower production cost. To design steel with superior properties for industrial application, it is essential to understand the effect of microstructure and engineer it to fit the purpose. In this study, a counter intuitive strategy has used to reveal the mechanism of high carbon steel with ultrahard structure. High compact force has been used to produce a structure which has ceramic-like hardness without compensating the toughness significantly. The behaviour of high carbon low-alloy steel as the starting material under different stages of deformation has been studied to differentiate various deformation paths and microstructural transformation processes. Microscopy investigation by secondary electron microscopy, high-resolution electron backscattered diffraction (HR-EBSD) analysis and Transmission electron microscopy (TEM) showed that the key point to achieve ~75% increased hardness in this steel is through generation of nano-structured martensite of less than 50 nm grains size which can be formed due to high impact force. In this paper, we reveal a nano grained steel structure with excellent mechanical properties resulting from phase transformation, uniform dislocation distribution, grain refinement and recrystallization.

## Introduction

High carbon steel is popular for its high hardness and good abrasion resistance. The toughness and abrasion resistance of low-alloyed high carbon steel can be enhanced by microstructural engineering to have martensite and retained austenite structure at the same time^1^. Meta-stable retained austenite phase in this grade of steel has the potential to transform to a more stable martensitic phase. This transformation happens by passing the phase transformation barrier energy through cooperative shear movement of atoms, upon application of stress^1–4^. This type of stress and strain induced phase transformation could be beneficial when the material is subjected to wear and abrasive condition and can absorb the applied energy and forms a new phase which has higher hardness^1,5^.

Stress induced phase transformation can be coupled with formation of new grains with smaller size which will increase the steel’s hardness and its toughness^6–8^. To enhance the mechanical properties, use of extra alloying elements is not always cost effective for low cost application as the price of cobalt, titanium, chromium, and manganese is tending to increase. Three main deformation mechanism of steel which include, transformation-induced plasticity (TRIP), twinning–induced plasticity (TWIP) and dislocation plasticity^9–11^, was incorporated in this study in combination of recrystallization which helped to achieve high hardness, but the mechanism did not reduce the toughness significantly. Each of these mechanisms was used in the past to develop different classes of steel, such as transformation-induced-plasticity steels with metastable austenite^1^ and twinning-induced-plasticity steels^12^ with stable austenite for high strength, high ductility and high energy absorption. But all these types of steels require very high alloying elements and hence are not cost efficient for low cost industrial application, new approach is required to strengthen the steel structure without adding excessive alloying element in the steel^13^. Refining the grains are considered as the key strengthening mechanism in the high strength steel as this phenomenon can increase the yield strength and toughness^14^. These kind of strengthening mechanisms have been applied to control the microstructures of modern high-strength steels which have expensive alloying or micro-alloying elements. Traditionally, the thermo-mechanical deformation processes are applied to refine the microstructure, which could produce the grain sizes of around 2 to 5 µm^15–17^. The aim to achieve the ultra-fine grains in steels having the grain size below 1 µm have been reported by many studies^18^. Severe plastic deformation in room temperature or elevated temperature is another pathway to achieve ultra-fine grains in steel^19–21^. A wide range of techniques have been reported to generate ultrafine grains in austenitic, ferritic or martensitic steels, mostly implemented in low carbon steel or plain steel^18^. However, none of the studies reported the grain refinement up to the nano level in case of low alloyed high carbon steel which could attract the interest of many industries for their superior hardness and strength.

The aim of this study is to reveal the micro mechanisms in low alloyed the high carbon steel associated to generate a new grade of steel with ceramic-like hardness and metal-like toughness by activating different deformation mechanism at high impact force and at the same time by successfully producing ultra-fine grains with low dislocation density through recrystallization due to generated heat. To investigate the new microstructures achieved by impact deformation and the associated mechanical behaviours at different load we have used secondary electron microscope (SEM), nano indentation, high resolution Electron backscattering diffraction (HR EBSD), Transmission Kikuchi Diffraction (TKD), Transmission electron microscope (TEM) and Focused ion beam (FIB) sectioning. Identifying the behaviour of this grade of steel, phase transformation as well as microstructural evolution of deformed phases down to the nanometre level is a vital aspect to characterising the low cost low alloyed high carbon steel as a potential new grade of steel for extreme operational conditions.

## Material and Method

A commercial grade of high-carbon steel, 0.89C–0.9Mn–0.6Cr (in wt. %), has been investigated in this research to study the microstructural evolution during solid state phase transition. The samples were at fully austenitic state at 1200 °C and then quenched in water to have the martensitic structure with significant amount of retained austenite. Compression deformation and impact deformation has been used to investigate the effect of activating different deformation mechanisms on microstructure and mechanical properties of this steel. A standardise static compression test has been used to investigate the effect of low strain rate and bulk deformation through a standard compressive load. The schematic for the drop ball test has been provided in the supplementary (Fig. 2s). The standardise compression test was followed as the previous research^1^. A dynamic impact test was used to investigating the localised deformation at high strain rate. To investigate impact deformation and applying high load in a fraction of second, a drop ball test was used. The free-falling balls were striking the fixed 20 mm thick sample with a value of force and velocity of 78.71 N and 12.78 m/s. 6 balls were dropped continuously to transform the base steel material into nanocrystalline steel. This velocity and force can be adjusted by changing the impactor’s weight and height to adjust the strain rate required for the experiment (Fig. 2s). During drop ball testing, high strain rate and temperature rise can occur within a very short time^22^ and also the cooling rate would be very fast which is reason behind the nano crystalline martensite formation^23^. High strain rate and a short annealing process due to the rapid temperature rise play an important role in the phase transformation and grain refinement of the steel. The cross-sectional specimen of the sample was cut from 1–3 mm depth from the surface of the compressed and the impacted sample for the microstructural observation. High resolution backscattered electron-based orientation microscopy investigation of stress induced samples was conducted by an Oxford system attached with a Carl Zeiss AURIGA CrossBeam field emission gun scanning electron microscopy (FEG SEM) workstation. The material preparation for the EBSD is provided in the supplementary. The orientation data were collected under the accelerating voltage of 20 kV, at 2 × 2 binning mode and at a step size of 50 nm. For the nano grained steel, we performed a Transmission Kikuchi Diffraction (TKD) of the FIB milled sample where the step size was 5 nm. The microstructure of the deformed sample was also observed using a TEM equipped with a field emission gun (Philips CM 200, Netherlands) after preparing by using a dual beam FIB (FEI xT Nova Nanolab 200, USA), and the thickness of the specimens was estimated to be ~100 nm.The cross-sectional morphology of the deformed sample has demonstrated phase transformation and grain refinement as a phenomenon of plastic deformation. SEM, EBSD and TEM analysis revealed the development of nano structured grains which have been discussed broadly in this research. The nano hardness of the steels at different stages of deformation was measured by a TI 900 Hysitron Tribolab system in load control mode with a Berkovich three-sided pyramidal diamond tip indenter (nominal angle of 65.3° and radius of 200 nm). Conventional micro Vickers hardness tests were also performed to obtain the hardness at 0.2 HV load.

**Figure 1:**
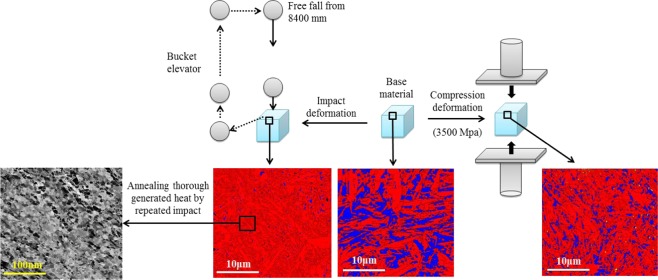
Schematic diagram of the deformation experiments. In EBSD diagrams, red is for martensite and blue is for retained austenite.

Figure 1 shows two main classes of deformation experiment used in this study. These two routes of deformation were chosen to activate different deformation mechanism, control the heat generation through deformation and control the microstructural changes. In pursuit of excellent enhancements in hardness and strength of metal, we proposed a new approach to convert the steel microstructure into the nano grained martensitic structure by activating three deformation mechanism and recrystallization which can produce nano grains with low dislocation density and ceramic like hardness.

**Figure 2:**
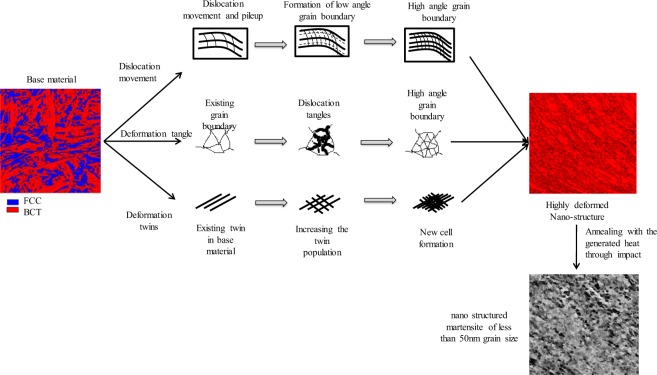
Deforma tion mechanisms activated during impact deformation. After impact deformation which contains explosive recrystallized martensitic structure.

In this study we have investigated various deformation mechanisms; dislocation movement, dislocation tangles and deformation twins which are summarised in Fig. 2. By applying different deformation modes, different deformation mechanisms will be activated. In the case of impact deformation, which was our counterintuitive strategy for producing ultrahard steel, the following evolutionary steps will occur. This will produce a dislocation free nano-grain structure. These steps involved are explained in detail in the next section of the paper.

(i) Base material contains large austenite and martensite grains and ~35% of the microstructure is austenite phase;
(ii) At the early stage of impact deformation, the austenite phase transforms to martensitic phase by compression deformation. This transformation happens through providing the necessary energy for transformation to austenitic phase via deformation. This transformation increases with the increasing strain rates and a subsequent grain refinement occurs. A reduction in the fraction of retained austenite occurs, comparing to an uncompressed sample. With the increased strain rates the reduction of retained austenite becomes more apparent.
(iii) Deformation and phase transformation both generate high dislocation density in metals irrespective of the phases which might lead to grain refinement in steels because of the formation of high-angle grain boundaries by rearrangement of intensive dislocations.
(iv) As the strain rate increases by the impact deformation, grain refinement takes place in various ways. The cross-slip of dislocation and formation of sub-cells transform to cells and refine the grain. New dislocation tangles and cells formed by increasing the strain rate which consequently forms new grains.
(v) Deformation and stress induce the twinning effect. At the low strain rate twin boundaries are parallel to each other and they subdivide the coarse grain. This phenomenon can occur in both the phases. At the high strain rate, intersections of twin boundaries in two directions results in more division. After several impacts it is predicted that with increasing stress and strain the multiplication of deformation twins imposes more twin twin intersections, and the micro structural refinement occurs with irregularly shaped grains and large misorientations. This phenomenon is also evident from the literature^24^.
(xvi) Through the generated heat by impact deformation, refined grains will be explosively recrystallised to produce dislocation free nano grained structure.

The nano grained layer can be created with single or multiple impacts depending on the impact force and angle and impact energy intensity which influences the thickness of the layer.

In this study, we prepared four different steel samples at various stage of deformation and characterized them in order to investigate the formation mechanism and properties of this newly developed steel. The steels that we explored and investigated the performance are, base material; after compression deformation under 3500 MPa and 10^–1^/s strain rates of static load; after impact deformation at 10^1^/s by one cycle; and after impact deformation within situ annealing by 6 cycles.

## Results and Discussion

### Microstructural evolution

**Figure 3:**
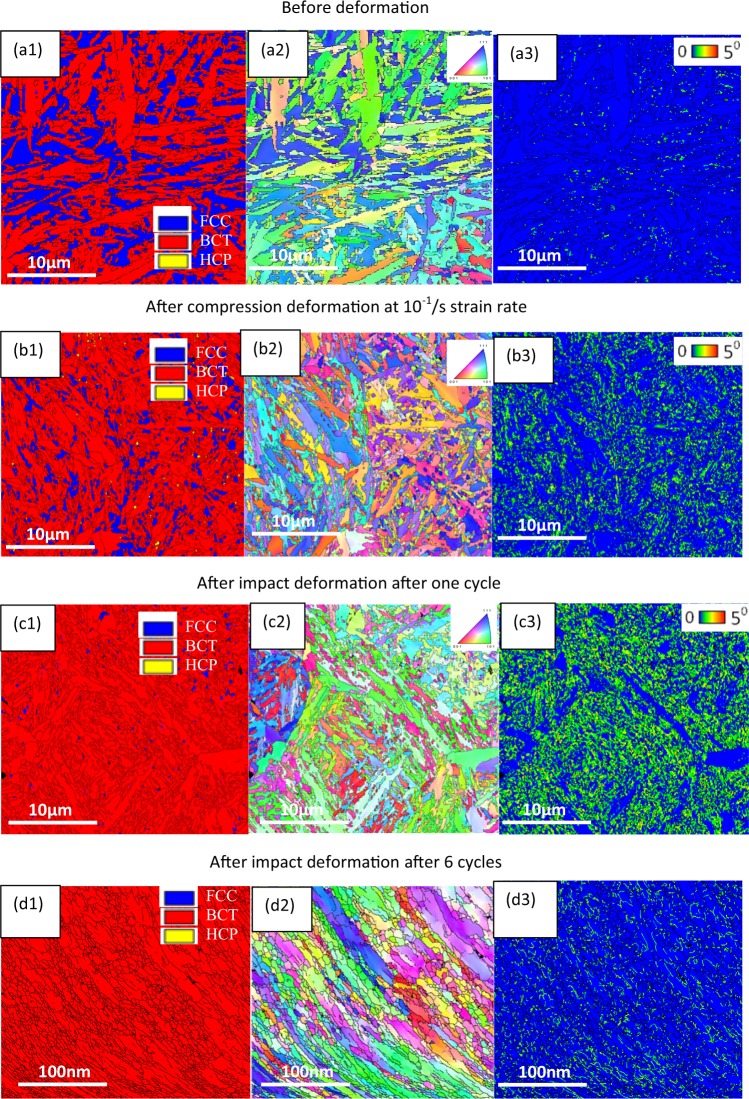
(a1) Phase map of base material (a2) IPF map of base material (a3) KAM map of base material (b1) phase map of steel after compression test (b2) IPF map of steel after compression test (b3) KAM map of steel after compression test (c1) phase map of steel after impact test (c2) IPF map of steel after impact test (c3) KAM map of steel after impact test. (d1) Phase map of steel after impact test and *in-situ* recrystallisation (d2) IPF map of steel after impact test and *in-situ* recrystallisation (d3) KAM map of steel after impact test and *in-situ* recrystallisation.

We have investigated all four steel samples using EBSD scanning to identify the changes in the grain size, grain orientation and dislocation density at different stage of deformation. The results were summarised in Fig. 3. As received dual-phase steel which contain martensite and retained austenite, exhibits a mixture of plate and lath martensite with a considerable amount of retained austenite (~35%) before deformation, Fig. 3a. The plate and lath martensite are indicated in the Supplementary Fig. 1s. Figure 3b shows the microstructures of the steel after plastic deformation which have been obtained through a compression process with compression load of 3500 MPa and strain rate of ~10^−1^/s. At the maximum static compression at room temperature (3500 MPa), the amount of retained austenite was reduced significantly (~10%). After this stage, the increased compressive load did not have any effect on the retained austenite and phase transformation which was revealed in our previous works^1,25^. Refined structure and increased grain boundaries act as a barrier to dislocation movements and hence to further phase transformation. Moreover, in static compression, due to low strain rate, the amount of heat generated is not adequate for recrystallisation to occur. In order to apply a high load in a fraction of a second, we have used the drop-ball test. During the impact test, when the free ball strikes the specimen repeatedly, the microstructure of the specimen transforms into a highly deformed martensitic structure. The austenite of the dual phase structure reduces by ~3–4%, Fig. 3c. If this drop-ball test continues for a several cycles, the generated heat will promote explosive recrystallisation phenomena which generate a dislocation free nano-grain martensitic structure, Fig. 3d. For intermediate strain rates (>1 × 10^−1^ s^−1^) the process is considered to be adiabatic in nature. Thus, based on the conservation of energy, the temperature for a single impact between the balls can be calculated by converting the impact energy into the heat energy considering no heat loss. From the given boundary conditions for FE modelling, the rise in temperature for a single impact is calculated to be 211.65 °C. Although the temperature increases due to multiple impacts using FE is not possible, it is assumed based on the single impact calculation presented here that the temperature rise can be estimated to be >750 °C. This argument is compatible with the previous finding where the researcher claimed that the temperature rise during high strain rate impact test can reach above 900 °C very rapidly^26^.

From the EBSD data. the kernel average misorientation (KAM) was obtained. The KAM is captured from the average misorientation in the microstructure surround a measurement point comparing with a defined set of the nearest neighbouring points^27^. Consequently, higher KAM value is an indicative of the higher dislocation density. In the current measurement, neighbouring pixels were considered having the misorientation angle less than 5° to exclude other boundaries, such as, grain boundary. The KAM maps display the change in dislocation content in the grain structure during different strain rates. The misorientation displays a shift in the distribution toward higher local misorientations as the strain rate increases. Increased misorientation angle is an indication of more dislocation density in the structure^28^. The KAM of the undeformed steel sample was found to be negligible as shown in Fig. 3a3, hence it is an indication of low dislocation density. The misorientation near some of the austenite-martensite grain boundaries shows a shift in the distribution towards higher local misorientations in the base material. After compression deformation the misorientation angle gradually increases, Fig. 3b3, and creates new grain boundaries but they are much fewer compare to impact deformation, Fig. 3c3. When impact deformation was applied, the misorientation angle increased drastically; hence the population of high energy point which can act as recrystallization point during recrystallisation turn out to be massive. At this stage with heat generated *in-situ* through repeated impacts, explosive recrystallization will produce a structure with very low misorientation angle, Fig. 3d3. The grain size was detected from few nano meters to less than 50 nm.

### Mechanisms of grain refinement

It is possible to create nano grains in the close vicinity of impact regions through the deformation mechanism and rapid temperature rise in the dropped ball^29,30^. Based on the calculated elastic and plastic strain from the FE modelling and the time required for impact, the obtained range of strain rates varied from 1.18 × 10^−1^ to 1.86 × 10^−1^ s^−1^ for a single impact and the generated compressive stress was ~3000 MPa. In this process, grain refinement will take place through three main mechanisms.

#### Dislocation formation

**Figure 4:**
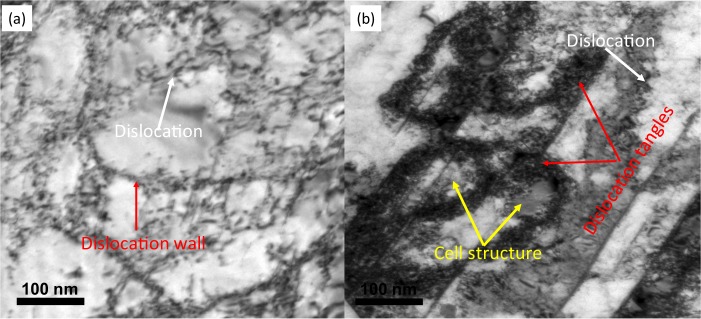
(**a**) Dislocation after compression at strain rate of ~10^-1^/s and (**b**) dislocation by impact deformation after one cycle.

By applying the load, dislocations start to form in the structure (Fig. 4a) and when the load increases, cross-slip of dislocations happens in between the different slip system^31^. This cross-slipping of dislocations promotes the formation of sub-cells with low-angle grain boundaries (Fig. 4b). By increasing the dislocation population, these sub-cells will form new cells and grain refinement will take place^32^. But these grains contain high dislocation density which will transform to dislocation free nano-grains through *in-situ* recrystallization. The kernel average misorientation (KAM) in Fig. 3a3,b3,c3,d3 are direct evidence of these steps.

Formation of dislocation and dislocation movement during deformation does not necessarily happen through arrays of dislocation but always there is a big portion of dislocations that are generated which randomly move and produce dislocation tangles^32^. If the number of dislocations in dislocation tangles increases, it will generate cells and grain refinement will take place (Fig. 4b). Figure 4a,b is the representative of this phenomena. In Fig. 4a, after compression deformation, the dislocation and dislocation wall are clearly visible. In Fig. 4b, after impact, these dislocations are accumulated and forms high density dislocation tangle. The cell structure confined by the dislocation is clearly visible in the Fig. 4b. Similar to the dislocation movement mechanism, generated grains by this process contain high dislocation density and after *in-situ* recrystallisation dislocation free nano-grains will form.

#### Deformation twin formation

The interaction of the glide dislocation and twin boundaries has a vital role in strengthening the metals and alloys. Presence of twins ceases the slip bands’ propagation and acts like the grain boundary which is an obstacle to the strain propagation^33^. It is believed that the slip is the dominant factor in plastically deformed metals and alloys which have the stacking fault energy in the range of medium to high. The metals with low SFE is favourable for mechanical twinning at high strain rate^12^. Increase in the strain rate will increase the twin density but decrease the twin thickness which has been proven in the observation of our previous study^4^. Formation of mechanical twins creates the twin boundary which is viewed as a twist boundary (60° (111)) or tilt boundary (75° (110)), is a large angle boundary. The large angle boundaries subdivide the deformed grains. Deformation twins are formed in parallel sets of twin boundaries. When the high strain rate is induced, many parallel sets of twin form at different directions^34^. When more than one set of twin system are activated to accommodate the deformation, different shapes of blocks are created by the intersection of the twin sets.

**Figure 5:**
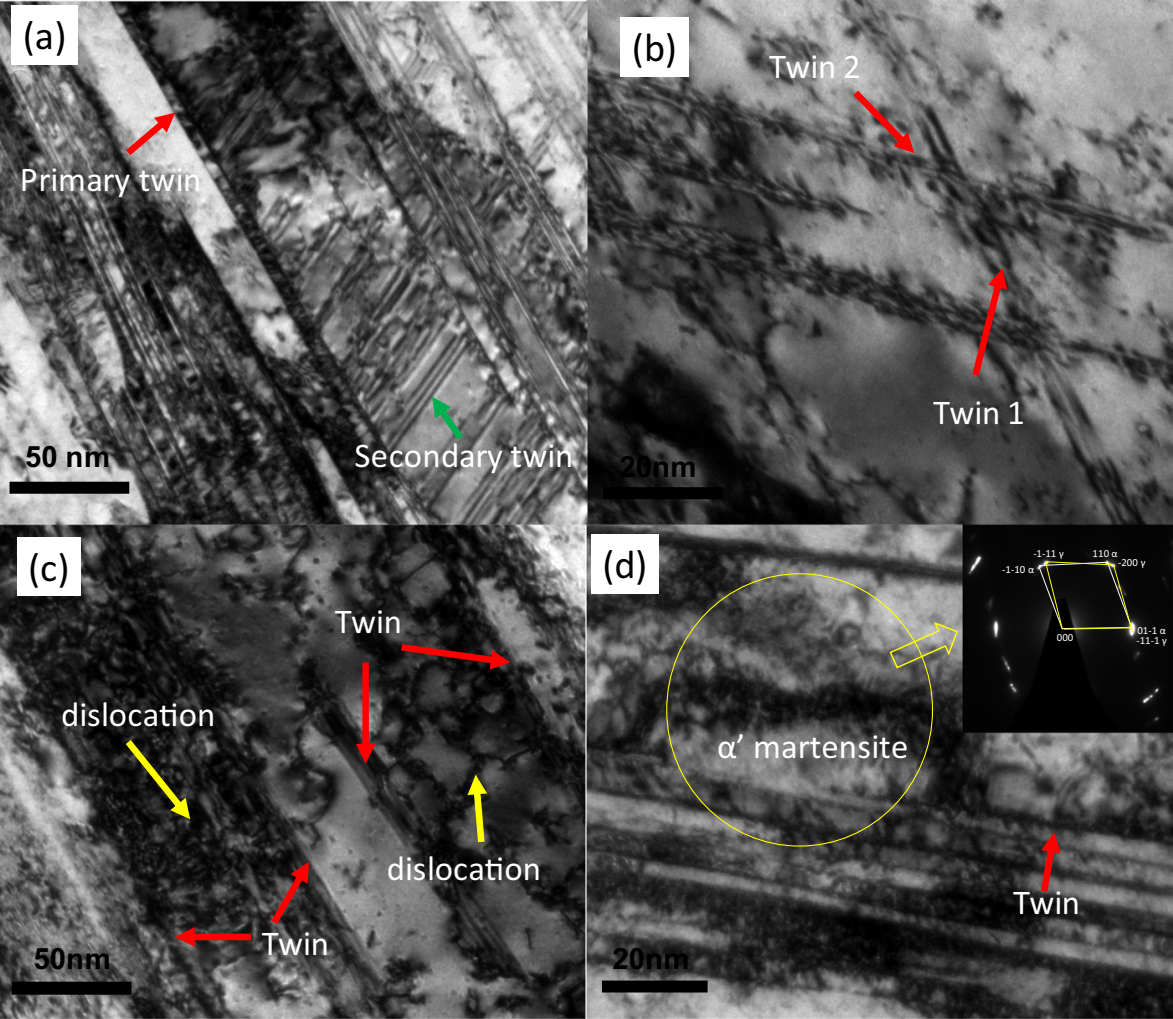
The TEM bright field image is showing twinning in blocky austenite grains after compression test at ~10^−1^/s. (**a**) Both primary and secondary twin system visible. (**b**) Twins formed at different direction. (**c**) Dislocation twin interaction. (**d**) Nucleation of martensite in the intersection of twins where the SAED diffraction pattern of the yellow circled area is showing K–S OR relation between α’ and γ.

In order to investigate the deformation twinning in more detail, twins in deformed sample was analysed. The bright field image of the TEM micrograph in Fig. 5a is showing very dense mechanical twins at high strain rate (10^−1^/s). Both the primary and secondary twins’ formation are evident in the deformed grains. The twins are forming in different direction in Fig. 5b. The intersection of twin bundles in some austenite grains may provide the potential nucleation site for martensite formation. This phenomenon is evident from Fig. 5d where newly formed martensite co exits with the austenitic matrix and the SAED patterns are following Kurdjumov-Sachs orientation relationship. The formation of twinning can facilitate two things; on the one hand it can promote the deformation by adding extra slip systems, on the other hand, it can assist the transfer between present slip systems through dislocation-twin interactions^35^. High dislocation density was found between the twins which is observed in the Fig. 5c. The competing activity of twin and slip system can strengthen the structure.

**Figure 6:**
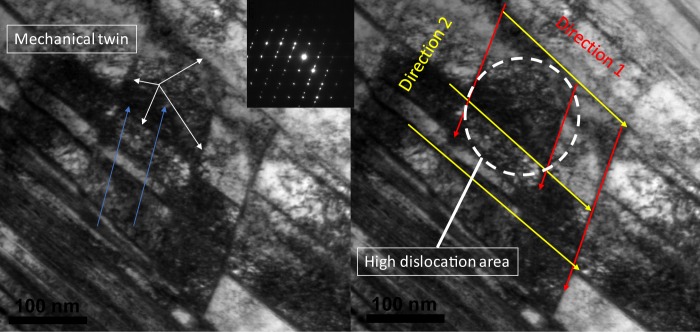
TEM image of the steel sample subjected to one impact. (**a**) The mechanical twin formation in the sample showing by white arrow, (**b**) different direction of the twin, direction one is in red and direction 2 is in yellow; the white circle is showing dislocation around the intersection of the twins.

As it shows in the Fig. 6a,b, during deformation, twins are formed through deformation and by increasing the population of twins, they will intersect in different direction and interact with dislocations. When this population and intersections increases, they will divide the grains into smaller sections which results in grain refinement. After several impacts, when the stress strain increases, the multiplication of deformation twins imposes additional twin twin intersections. As a result, the structure is refined into unevenly shaped grains which have comparatively larger misorientations^34^.

The chemical concentration and the stacking fault energy (SFE) may affect the formation of mechanical twins and the phase transformation process. SFE is related to the chemical composition, such as, C and Mn content and therefore an elementary analysis in the retained austenite was conducted using EPMA. Both C and Mn elements are the strong austenite stabilizers^3,36^. The SFE depends on the chemical composition and temperature. It has a close relationship with the martensitic transformation of the retained austenite and twinning and can be calculated by the following equation^37^:

1 $$SFE=2\rho \Delta G+2\chi $$

where ρ is the molar surface density along the austenite close-packed plane, Δ*G* is the molar Gibbs energy of transformation and 2*x* is the interfacial energy which is 14 *mJ*/*m*^2^.

**Figure 7:**
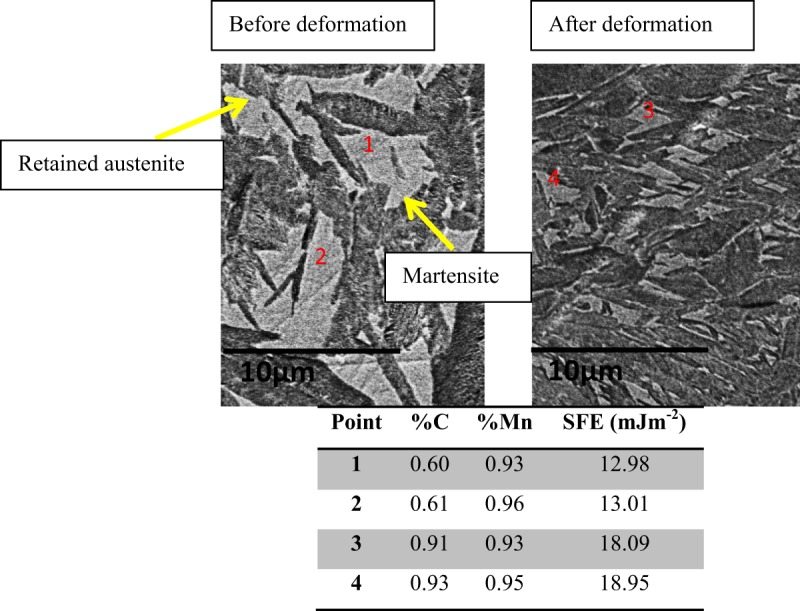
The percentage of C and Mn by EPMA investigation and calculated Stacking Fault Energy of designated points in retained austenite. Point 1 & 2 are in sample before deformation and point 3 & 4 are in deformed sample.

There is two different phase stability of retained austenite; one is chemical stability, and the other is mechanical stability. The stability of austenite is increased when the concentration of austenite stabilizer is higher (C, Mn), and the grain size is smaller^38^. In case of chemical composition, all austenite grains should show a similar chemical concentration if the austenite grains were originated in the same conditions. In this work, all austenite grains were originated from the base-materials. However, if the austenite grains have different grain size in the same sample, the bigger austenite grains will have less C enrichment due to the fact that usually they do not surrounded by martensite which could let the C enrichment^3,12^. Therefore, if the austenite grain is larger, it will have comparatively less C content, and consequently have lower mechanical stability within the base steel^12,25^. Therefore, they shall transform at a low stress and strain level compared to the smaller austenite grain surrounded by martensite^25^. However, if the chemical composition is same for all austenite, another factor that will affect the phase transformation is the grain size of retained austenite. When the austenite grains have same chemical composition with different grain size, smaller austenite grains will be mechanically more stable^1^. This may be due to two reasons: (1) when the grain size of the retained austenite increases the strain energy decrease because of the nucleation of single martensite variant^39^; (2) when the austenite grain is smaller, it has less chances for the multi-variants nucleation and can reduces the strain energy more^40^. Therefore, different grain size contributes to different levels of stability of retained austenite^25^. In this study, EPMA was used to know the chemical concentration of C and Mn. Mn content does not vary among the austenite grains, but the C content varies a little bit which can cause varying SFE^1,12,25^. We determined the SFE of austenite grains in both deformed (after compression) and undeformed samples which reveals that the bigger austenite grains have average SFE of ~13 mJm^−2^, while, the smaller austenite grains are C enriched and have a much higher SFE, ~18.65 mJm^−2^ (Fig. 7). It has been reported that the formation of mechanical twins is suitable for the SFE between 12 mJm^−2^ to 35 mJm^−2^ while the martensitic transformation of retained austenite is favoured for the SFE below 18 mJm^−2^. In case of deformed sample, more smaller grains are there which is more stable and need more energy to transform. This stability could come from the smaller grains and very dense twins in the untransformed austenite (Fig. 5).

### Microstructure of nano grained steel

**Figure 8:**
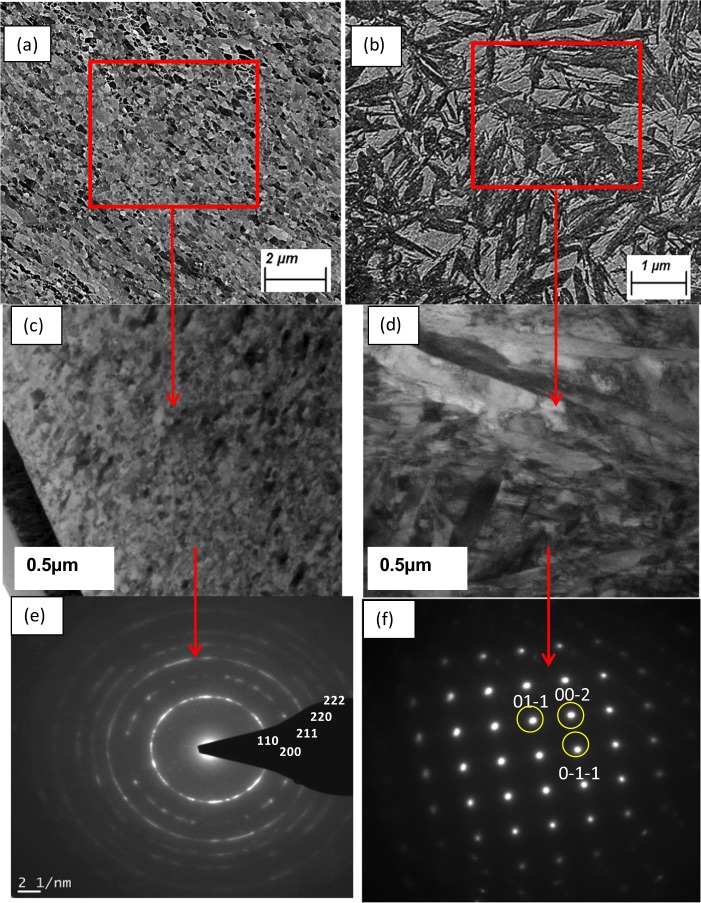
Electron channelling contrast imaging of steel shows (**a**) nano structure after impact and (**b**) base material. TEM bright field image of **(c**) steel after impact deformation and *in-situ* recrystallisation (**d**) base material. SAED pattern of (**e**) steel after impact deformation and *in-situ* recrystallisation (**f**) base material is showing the diffraction pattern for martensite.

The high-resolution Transmission emission microscopy-based technique was used to identify the refined grain structure after impact and *in-situ* recrystallisation and was compared with the sample, which was not subjected to impact test, the base material. Figure 8b,d,f is the SEM, TEM images and SAED pattern of the base material. Figure 8a,c,e is the SEM, TEM images and SAED pattern of the nano structured steel after impact test. This imaging will help to understand the deformation mechanism of dynamic impact on the steel sample. Selective area electron diffraction (SAED) patterns of the different zones are presented in Fig. 8e,f. In the SAED image of the nano grained zone, two dramatic differences were observed compared to the original metal matrix:

1. A great structural refinement (SAED ring pattern only form when fine structure is involved) occurred due to the deformation compared to the base material structure where a clear cubic structure is visible.
2. The SAED patterns in nano grains were indexed and found to correspond to the α’ (BCT) martensite phase only.

Crystallization of the nano grains depend on the relation between the rate of dynamic recovery and the mobility of the grain boundaries of metal^41^. Continuous transformation of high angle grain boundaries was identified due to the development of geometrically necessary boundaries in the recrystallization process. Diverse combinations of slip systems develop within the micro volumes which are separated by the high angle boundaries. Because of the continuous deformation, misorientations within the micro volumes increases and eventually transform into high angle boundaries. Greater strains lead to the formation of ultrafine grain microstructures. It can thus be concluded that initially the subgrains of martensite phase contain low misorientation angle (less than 5°) and offered low to moderate strains with high dislocation density that gradually transformed into ultrafine grains of martensite by the creation of high angle boundaries with negligible dislocation density at larger strains. The stress induced grain refinement, phase transformation and mechanical twinning can alter the mechanical property, especially hardness of high carbon steel.

### Properties of nano-grained martensitic structure

From the deformation mechanism of this low cost low alloyed high carbon steel, we have successfully realised that it is possible to produce a new grade of steel which has high hardness and good toughness by engineering the microstructure as it summarised in Fig. 2. Impact defamation can activate all three-deformation mechanism and after *in-situ* recrystallization uniform grain with very fine size can be produced. These uniform grains can elevate the hardness of the nano grained steel and give it a ceramic like hardness.

**Figure 9:**
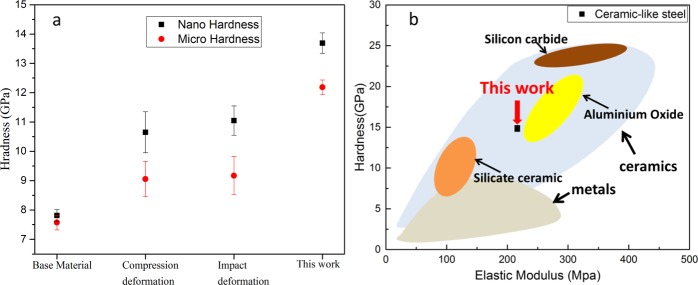
(**a**) Nano hardness and micro hardness of studied steel before deformation and after different stages of plastic deformation and after *in-situ* recrystallization. (**b**) Hardness and elastic modulus comparison of the nano grained steel in this work with ceramic and metals.

To investigate the hardness of each sample after deformation, we performed nano indentation hardness test. The base material has the hardness around 7.87 GPa in the nano hardness measurement and 7.58 GPa in the microhardness measurement. The average hardness after compression deformation reached to ~10.65 GPa, in the nano hardness measurement and after impact deformation reached to ~11.05 GPa but after *in-situ* recrystallization and formation of nano grained structure, the average nano hardness reached as high as ~13.69 GPa which is ~75% higher than the base material (Fig. 9a). The hardness of the nano grain might be attributed to two effects, the Hall-Petch effect and dislocation strengthening. Apart from the strengthening effect due to the grain refinement, increased dislocation density and deformation twinning also contribute to the improvement in the material hardness and strength^21^. Conventional micro Vickers hardness tests were also performed to obtain the hardness at 0.2 HV load. The results of the micro hardness test are the average of 10 data point. The error bars are showing the 95% confidence intervals (Fig. 9a). The nano-hardness measurement has higher values compared to the micro-hardness measurements and this information is consistent with the results. It has been reported that the nano-hardness data show 10–30% higher values than the Vickers hardness data^42^. A general comparison of the nano hardness of different materials with the nano hardness on this work has been demonstrated in Fig. 9b.

**Figure 10:**
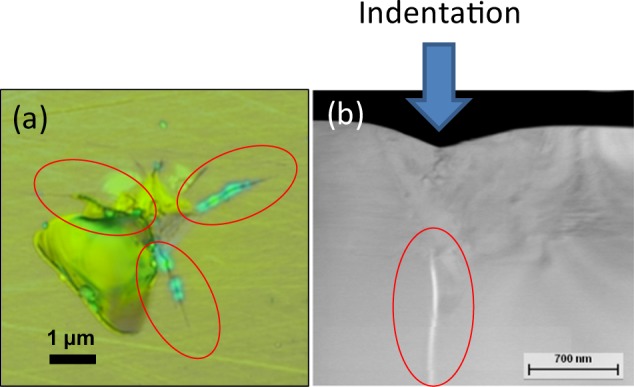
(**a**) Optical microscopy image of the post intonation area. (**b**) TEM image of the cross section of the indentation is revealing the crack morphology.

For further evaluation, we determined the nano indentation fracture toughness of the ceramic like steel and compared it with different ceramic materials and high carbon steel. Nanoindentations were carried out on the polished samples by a berkovich diamond indenter tip under various peak loads ranging from 10mN to 80mN. Force–displacement (P-h) curves for indents which did not create cracks were used to calculate the nanohardness and elastic modulus. Post-indentation images were acquired immediately after indentation to measure the lengths of the cracks. An example of post indentation image is provided in the Fig. 10. Figure 10a,b confirms that a crack geometry has been introduced perfectly in the three corners of the berkovich diamond indenter and underneath the indentation. Fracture toughness was calculated using the following equation^43^:

2 $${K}_{c}=\alpha {[\frac{E}{H}]}^{\frac{1}{2}}[\frac{P}{{c}^{\frac{3}{2}}}]$$

where K_c_ = fracture toughness, E = Young’s modulus, H = hardness, P = peak applied load, C = average length of the radial cracks, and α = empirical constant; taken as 0.032 for a cube-corner tip.

**Figure 11:**
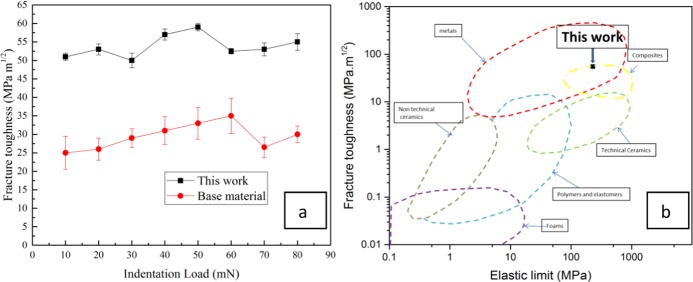
(**a**) Fracture toughness as a function of indentation load for nano-grained martensitic steel in this work (ceramic-like steel) and base material, (**b**) fracture toughness comparison of nano-grained martensitic steel in this work with ceramic, metals and other materials.

The indentations made by the indenter tip at a load higher than the crack threshold load. After the indentation, the samples have three radial cracks at the corner of the indent (Fig. 10a). The fracture toughness measurement by the nano indentation experiment at different peak loads are summarized in Fig. 11a. A fracture threshold of 9.8mN was identified below which no crack was observed in the sample with the indenter tip under the selected experimental conditions. The measured fracture toughness values suggest superior crack resistance comparing it with other hard materials (Fig. 11b). This is because, a certain energy is required for the fracture path to cross grain boundaries. Consequently, the more grain boundaries there are to cross, the higher the fracture toughness, for a given strength of metal. The grain refinement creates more grain boundaries and makes hindrance for the fracture path to travel. Thus, the nano grained structures increased the hardness without compromising much the toughness of the material. It should be noted that the quantitative analysis of this fracture toughness has been done for the comparison purpose only. The actual fracture toughness of the material may vary from these results.

The optical microscopy is showing that the uniformed nano grained structures are created in the impacted sample. The effective depth of this structure can vary from 1 mm to 2 mm. For the compressed sample, the effect was seen in the overall structure (Figs. 3s and 4s). A controlled impact deformation via drop ball test was performed to create this martensitic structure with the nano-grains through extreme deformation combined with *in situ* recrystallizations which is formed as a uniform layer. This structure has ultra-hardness with reasonable fracture toughness can facilitates the surface modification of bulk steel structure without additional alloying element and is suitable for modifying the surface properties of the low alloyed high carbon steel. The investigation of the fracture toughness by nano indentation cannot accurately identify the bulk property but it could represent the comparison of the fracture toughness between different materials. The previous study reported that the actual fracture toughness of dual phase high carbon steel varies from 34 to 45 MPa.m^−1/2 ^^44^. In our study we have got the fracture toughness of the base material ~2–5% less than this value by the nano indentation (Fig. 11). This observation reveals that the nano grained structure has achieved a superior fracture toughness. The study for the actual fracture toughness is exempted for the current investigation.

This result advocates that by generating nano structure through deformation induced phase transformation with multiple micro-mechanisms in the high carbon steel, ultra hardness without compensating the fracture toughness could be achieved.

## Conclusion

The development of materials with dual properties of ultra hardness and superior toughness has been a persistent challenge in manufacturing steel products. This study revealed that a fully recrystallized nanostructure with very high hardness without compromising the toughness can be fabricated with controlled deformation in low alloyed high-Carbon martensitic steel without adding any expensive alloying element. By altering the microstructure and dislocation distribution, transforming the phase stability to concurrently trigger multiple micro-mechanisms like formation of dislocation tangle and twin twin interaction induced new cell in the low alloyed high carbon steel can provide the observed enhancement in mechanical response for industrial application. Deformed steel consists of nano-grained martensitic structure where the grain size is less than ~50 nm with relatively very low dislocation density. This kind of structure is not present in the conventional engineering alloys such as dual-phase steel, pearlitic steel, or transformation induced plasticity (TRIP) steel. These strategies suggest the potential to redesign the steel with superior properties via microstructural engineering, while keeping it cost effective by not adding expensive alloying elements.

## Supplementary information

Supplementary Information

## Data Availability

The data that support the findings of this study are available from the corresponding author upon reasonable request
