# Supplementary material for: Revealing the mechanism of extraordinary hardness without compensating the toughness in a low alloyed high carbon steel
Source: Sci Rep. 2020 Jan 13;10:181. doi: 10.1038/s41598-019-55803-6 (PMC6957677; doi:10.1038/s41598-019-55803-6)
Supplement: Supplementary file 1 — Supplementary Information [file 41598_2019_55803_MOESM1_ESM.docx]

**Revealing the mechanism of extraordinary hardness without compensating the toughness in a low alloyed high carbon steel**

Rumana Hossain*, Farshid Pahlevani**, Veena Sahajwalla

*Centre for Sustainable Materials Research and Technology, School of Materials Science and Engineering, UNSW Sydney, Australia.*

** Corresponding Authors: E-mail address:* [*r.hossain@unsw.edu.au*](mailto:r.hossain@unsw.edu.au)

*** Corresponding Authors: E-mail address:* [*f.pahlevani@unsw.edu.au*](mailto:f.pahlevani@unsw.edu.au)

Supplementary:

**Specimen Preparation for microscopy**

The steel samples were then cut precisely using a Struers Accutom-50 diamond cutter at a very low speed. The samples were then hot mounted and fixed in a 30 mm diameter ring mould with 15 ml conductive Polyfast resin using a Struers CitoPress-20. The heating temperatures were set at 80 °C (pre-heating) and 180°C for 3 minutes of each temperature. The hot mounted samples were then cooled by water for 2 minutes. The metallographic polishing schedule is listed in Table 1s.

**Table 1s. 1 Metallographic polishing schedule used for the high carbon steel**

| Abrasive (P-[grade]) | 320 Grit (P-400) | 600 Grit (P-1200) | 800 Grit (P-2400) | 1200 Grit (P-4000) | 3 µm | 1 µm | 0.05 µm |
| --- | --- | --- | --- | --- | --- | --- | --- |
| Type | Silicon carbide | Silicon carbide | Silicon carbide | Silicon carbide | Polycrystalline diamond | Polycrystalline diamond | Colloidal suspension |
| Platen speed (rpm) | 300 | 300 | 300 | 300 | 150 | 150 | 150 |
| Time (min) | 2-5 | 2-5 | 2-5 | 2-5 | 10 | 10 | 5-10 |

The samples were ultrasonically cleaned for 5 minutes in an organic solvent prior to the next polishing each time. Finally, the sample was ion milled to remove the deformed layer induced by metallographic polishing. before the EBSD.

To prepare the TEM and TKD sample preparation the precision ion beam thinning, electropolishing and thinning with the focused ion beam coupled with SEM are used. The sample thickness is critical, and best results are achieved with comparatively thin samples of less than 100 nm thickness in case of high carbon steel.

**Base material**

Figure 1s EBSD pattern of the base material, the red is for martensite and the blue is for austenite. The white arrows are indication plate and lath martensite.

**Schematic of drop ball test**

A dynamic impact test was used to investigating the localised deformation at high strain rate. To investigate impact deformation and applying high load in a fraction of second, a drop ball test was used. The free-falling balls were striking the fixed 20 mm thick sample with a value of force and velocity of 78.71N and 12.78 m/s. 6 balls were dropped continuously to transform the base steel material into nanocrystalline steel. This velocity and force can be adjusted by changing the impactor’s weight and height to adjust the strain rate required for the experiment. During drop ball testing, high strain rate and temperature rise can occur within a very short time {Hossain, 2017 #32}. This information has been included in the supplementary.

Figure 2s Schematic diagram of the impact test

**Optical microscopy**

*Figure 3s The optical microscopy of the impacted sample showing the effective depth of nano grained steel.*

*Figure 4s Optical microscopy of the steels. (a) base material before compression; (b) material after compression at 2000 MPa.*

References:

1. Hossain, R., et al., *Stability of retained austenite in high carbon steel under compressive stress: an investigation from macro to nano scale.* Scientific reports, 2016. **6**.

2. Hossain, R., et al., *Hybrid structure of white layer in high carbon steel–Formation mechanism and its properties.* Scientific Reports, 2017. **7**: p. 13288.
